# Case Report: HHV8-positive multicentric Castleman disease in an HIV-positive patient :diagnostic challenges arising from atypical histology and the role of metagenomic sequencing

**DOI:** 10.3389/fonc.2026.1779973

**Published:** 2026-03-31

**Authors:** Wei Zhang, Rui Huang, Jing Yuan

**Affiliations:** Division of Infectious Diseases, Chongqing Public Health Medical Center, Chongqing, China

**Keywords:** diagnosis and treatment, HHV8, HIV, lymphoproliferative disorder, multicentric Castleman disease

## Abstract

**Background:**

Multicentric Castleman disease (MCD), especially the HHV8-positive subtype, is a rare lymphoproliferative disorder that presents considerable diagnostic and therapeutic difficulties, particularly among HIV-positive patients. The co-occurrence of other infections, such as syphilis, may further complicate its clinical picture and management.

**Case description:**

A 65-year-old man with well-controlled HIV presented with persistent fever, fatigue, and disseminated lymphadenopathy,. Through histopathological examination, molecular testing (including mNGS for HHV8), and PET-CT imaging, HHV8-positive MCD was diagnosed, along with latent syphilis. The patient was successfully treated with R-VP16 (rituximab and etoposide) for MCD and benzathine penicillin for syphilis, showing a positive clinical response. Throughout 36 months of continuous monitoring, the patient has maintained sustained complete remission with no evidence of disease recurrence.

**Conclusion:**

This case underscores the importance of considering HHV8-driven lymphoproliferative disorders in HIV patients with unexplained lymphadenopathy and systemic symptoms, particularly in HHV8-endemic regions. It also highlights the essential roles of advanced diagnostics and multidisciplinary management in such complex presentations. The favorable outcome demonstrates the effectiveness of timely and targeted treatment, though long-term follow-up remains necessary due to the potential for relapse or progression.

## Introduction

Multicentric Castleman disease (MCD) is a rare lymphoproliferative disorder marked by systemic inflammation, lymphadenopathy, and cytokine-mediated symptoms. It is frequently associated with human herpesvirus-8 (HHV8) infection, especially among people living with HIV (PLWH) (1). HHV8-positive MCD represents a distinct subtype within the Castleman disease spectrum, driven predominantly by viral interleukin-6 (vIL-6) and other cytokines. This leads to severe systemic manifestations and, if left untreated, carries a considerable risk of progression to lymphoma (2). Diagnosing HHV8-positive MCD in HIV patients remains challenging due to significant overlap in clinical and radiological features with lymphoma, reactive lymphadenopathy, and other opportunistic infections (3).

The coexistence of HIV, HHV8-positive MCD, and syphilis further complicates both diagnosis and management. Syphilis, a sexually transmitted infection caused by Treponema pallidum, can present with generalized lymphadenopathy and systemic symptoms that mimic MCD or lymphoma (4). In HIV-positive individuals, syphilis may show atypical serological responses and more rapid progression, requiring careful interpretation of laboratory results (5). The interaction between HIV, HHV8, and syphilis can worsen immune dysregulation, potentially leading to a more aggressive disease course and poorer outcomes if not addressed promptly (6).

This case illustrates the diagnostic difficulties and therapeutic approaches for HHV8-positive MCD in an HIV-positive patient with concurrent latent syphilis. The patient initially presented with painless lymphadenopathy, later developing fever and systemic symptoms, highlighting the need for a comprehensive diagnostic workup. This should include histopathology, molecular testing (such as mNGS for HHV8), and advanced imaging (e.g, PET-CT) (7). The patient responded well to combined therapy with R-VP16 (rituximab plus etoposide) for MCD and benzathine penicillin for syphilis, underscoring the value of targeted treatment in such complex presentations (8).

The rarity of HHV8-positive MCD, its diagnostic pitfalls, and the lack of standardized treatment protocols for HIV-associated cases emphasize the clinical relevance of this report. By detailing the diagnostic pathway, therapeutic decisions, and long-term follow-up, this case adds to the evolving literature on HHV8-driven lymphoproliferative disorders in immunocompromised hosts (9). Moreover, it stresses the importance of maintaining a high index of suspicion for MCD in HIV patients with persistent lymphadenopathy and systemic symptoms, particularly in regions where HHV8 is prevalent (3).

## Case presentation

### Patient information

A 65-year-old married male, office worker,diagnosed with HIV in 2011 (baseline CD4^+^ count: 84 cells/µl; initial viral load undocumented), was initially treated with AZT/3TC/NVP. He switched to bictegravir/emtricitabine/tenofovir alafenamide in May 2022. In March 2022, he developed painless lymphadenopathy in the cervical, axillary, and inguinal regions, with the largest node being chestnut-sized, firm, smooth, and mobile. There was no fever, erythema, discharge, or skin adhesion. The patient did not seek medical care initially.

### Clinical findings

**Figure 1:**
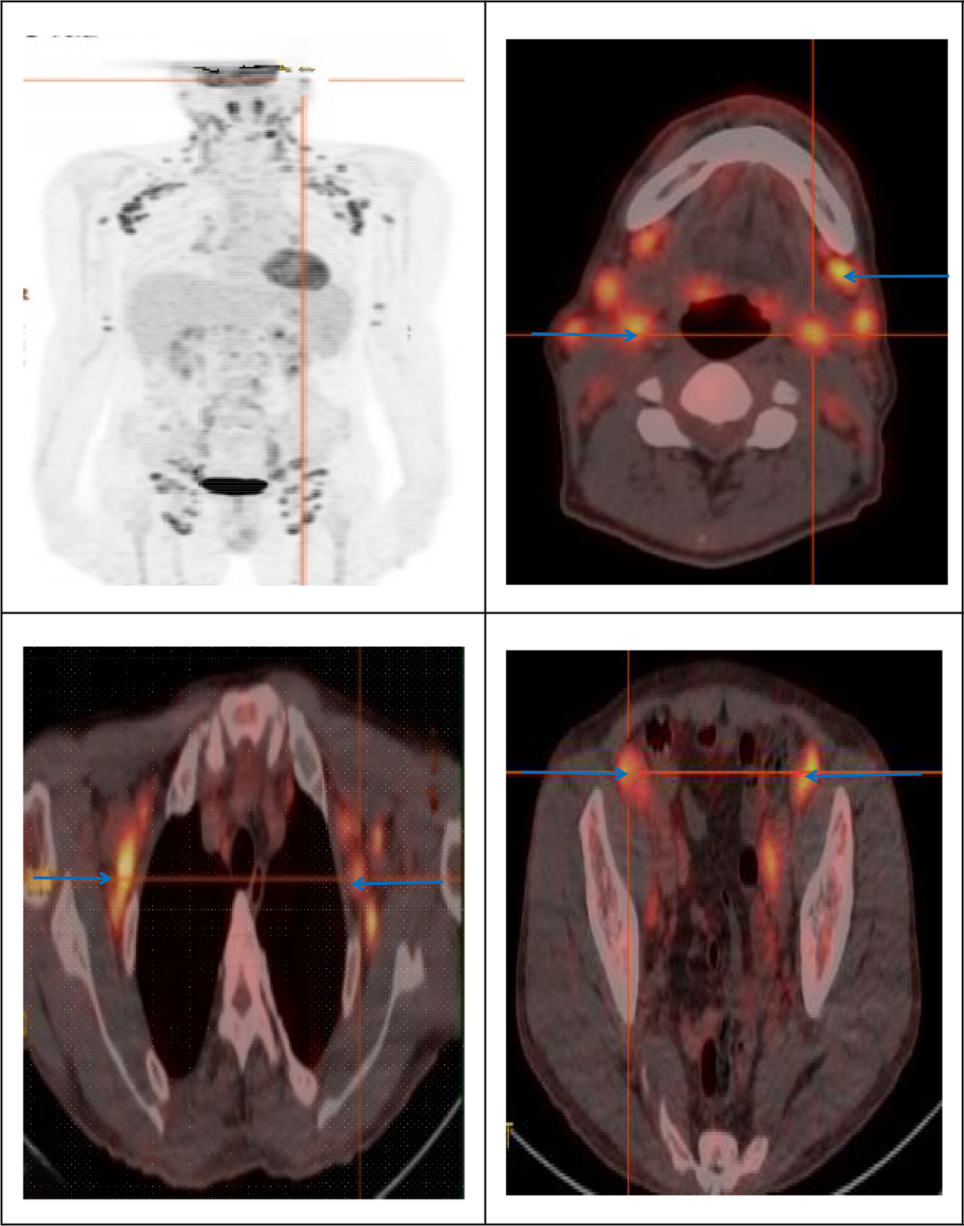
Before treatment, the patient’s pretreatment 18F-FDG PET imaging showed multiple sites of high uptake.

On June 18, 2022, he presented to Tianjin Second People’s Hospital with mildly enlarged cervical nodes. A biopsy on June 22 suggested reactive hyperplasia. PET-CT on June 29 revealed widespread lymphadenopathy with elevated FDG uptake, based on diffuse, symmetric nodal uptake and absence of focal destructive lesions, including bilateral cervical (max SUV 11.03), axillary (max SUV 10.03), thoracic, abdominal, and inguinal nodes(**Figure 1**). Although lymphoma was suspected, inflammatory changes were considered more likely. The patient received treatment with glucocorticoids. and was discharged with follow-up advice.

He returned on July 4 with new-onset fever (up to 39.5 °C), fatigue, anorexia, chest tightness, dyspnea, and progressive lymph node enlargement. Repeat lymph node puncture again indicated reactive proliferation. Bone marrow biopsy with histopathological examination and flow cytometry, which showed no lymphoma infiltration. Metagenomic next-generation sequencing (mNGS) of lymph node tissue detected HHV-8 and EBV. Symptoms improved with anti-infective and glucocorticoid therapy, and he was discharged after lymph node regression. On August 27, he was readmitted with recurrent high fever and worsening lymphadenopathy, prompting referral to our hospital.

Physical examination at admission showed enlarged, firm, non-tender, mobile lymph nodes in the cervical, axillary, and inguinal regions—the largest being egg-sized—without erythema or ulceration. Vital signs were stable, and systemic examination was otherwise normal.

### Diagnostic assessment

Lab tests showed a CD4^+^ count of 435 cells/µl, CD4/CD8 ratio of 0.44, and undetectable HIV viral load. Notable findings included thrombocytopenia (85×10^9^/L), mild anemia (Hb 118 g/L), and elevated inflammatory markers: ESR 69 mm/h, CRP 68.33 mg/L, and IL-6 21.86 pg/mL. Liver and kidney function were normal. Syphilis serology was positive (TPPA+, RPR 1:2). EBV IgG positive, EBV DNA negative. Chest CT suggested possible lymph node space-occupying lesions. Abdominal CT revealed splenomegaly, retroperitoneal lymphadenopathy, and a cystic lesion in the right inguinal region.

**Figure 2:**
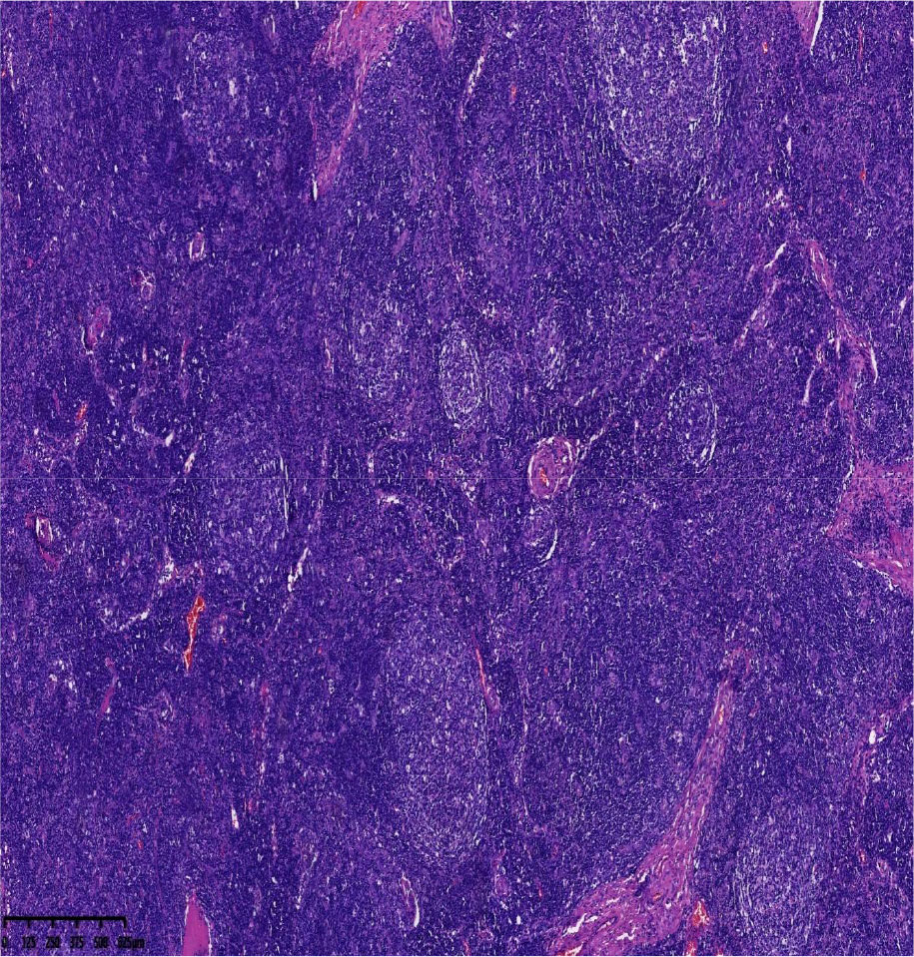
H&E stain,40. Histology showed follicular hyperplasia with mantle zone expansion exhibiting an “onion-skin”pattern, vascular proliferation.

**Figure 3:**
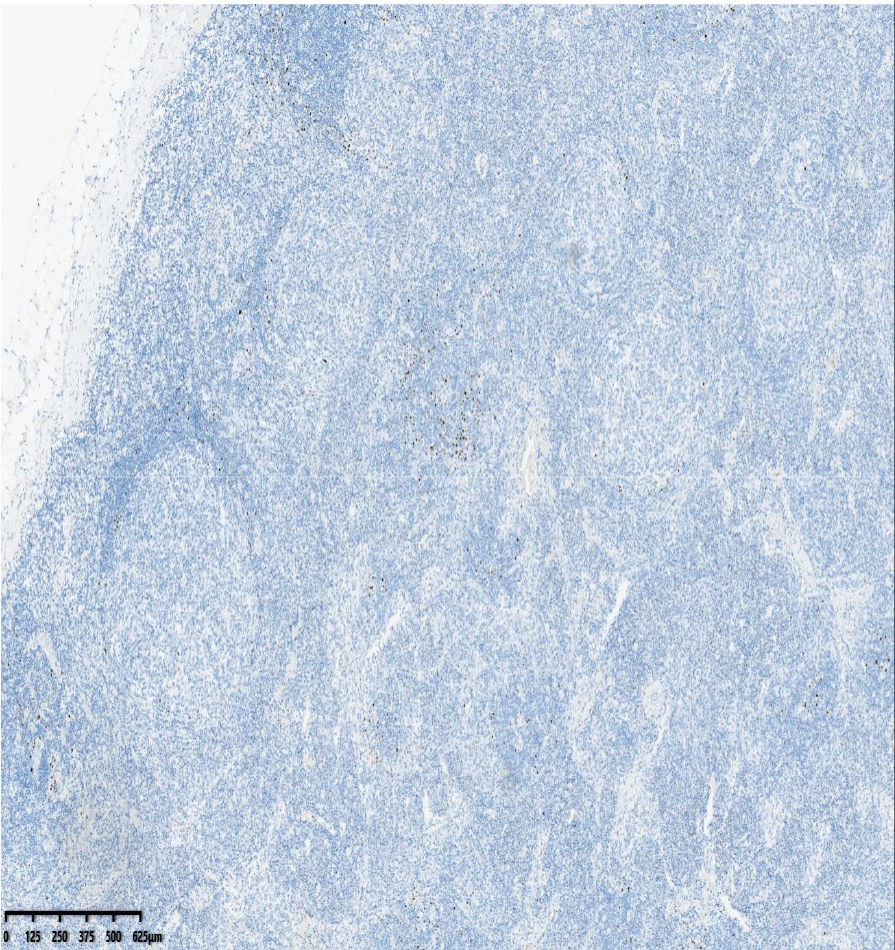
HHV-8 +,40x,HHV-8 positive.

After the results of the puncture biopsy could not confirm the diagnosis, a resection biopsy was eventually performed to make a definite diagnosis. Excisional biopsy of cervical and inguinal nodes confirmed the diagnosis: histology showed follicular hyperplasia with mantle zone expansion exhibiting an “onion-skin” pattern, vascular proliferation, and HHV-8 positivity on immunohistochemistry. These findings supported HHV-8^+^ multicentric Castleman disease, plasma cell type (**Figures 2**, **3**).

### Therapeutic intervention

The patient was diagnosed with HIV-associated HHV-8^+^ multicentric Castleman disease and latent syphilis. Treatment began on September 22, 2022, with four weekly cycles of rituximab (375 mg/m²) and etoposide (100 mg/m²). Syphilis was treated with benzathine penicillin G (2.4 million units IM weekly for three weeks).Persist in using bictegravir/emtricitabine/tenofovir alafenamide for HIV treatment.

### Follow-up and outcomes

**Figure 4:**
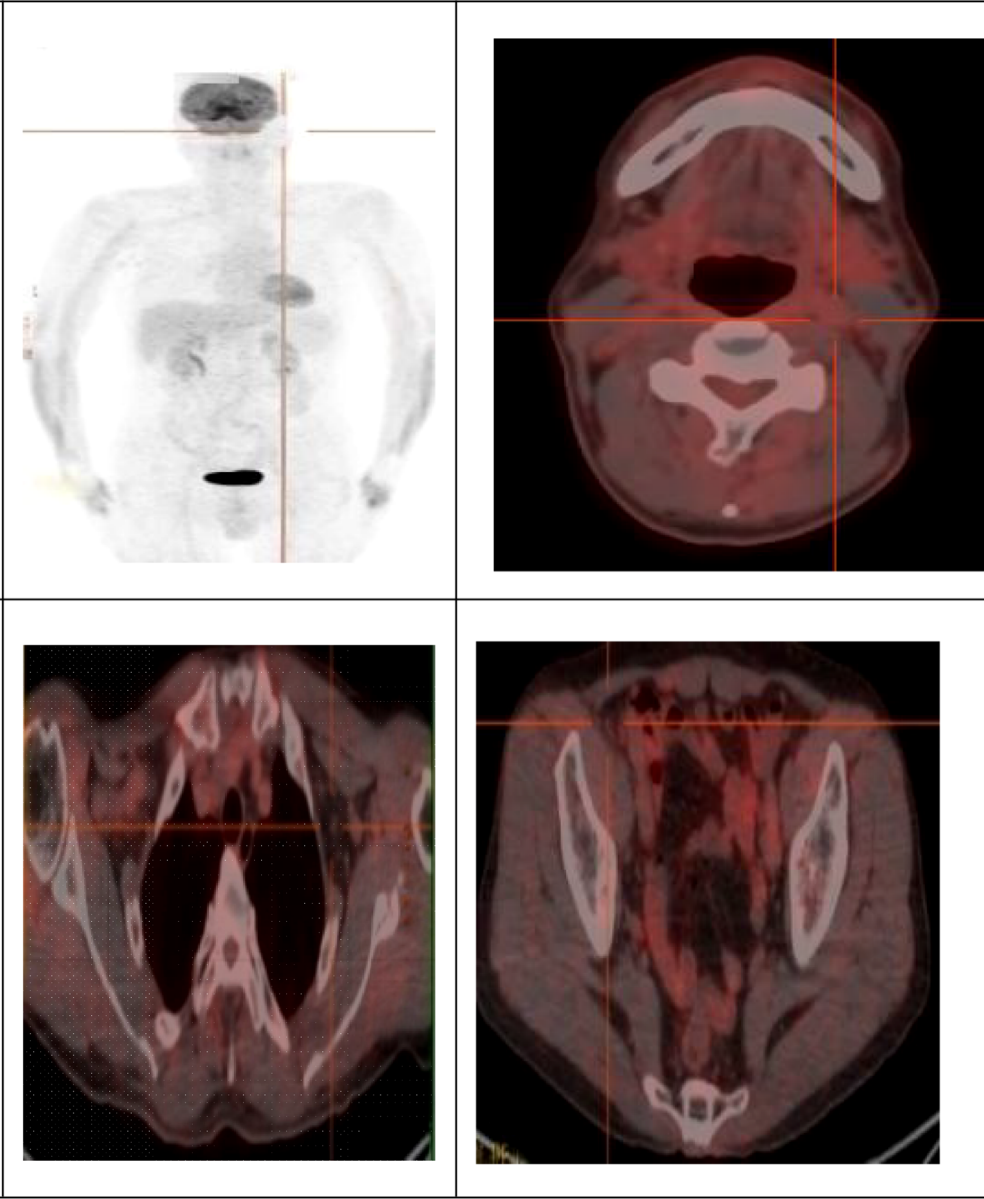
After treatment, 18F-FDG PET imaging showed that the lesions disappeared.

A follow-up PET-CT scan in January 2023 revealed significant therapeutic improvement, characterized by a marked reduction in the size and number of lymph nodes across all previously involved regions, along with substantially decreased FDG uptake (SUVmax 2.52, mean 1.76), indicating an excellent treatment response (**Figure 4**). Throughout 36 months of continuous monitoring, the patient has maintained sustained complete remission with no evidence of disease recurrence (**Table 1**).

**Table 1:** Timeline.

2011	The patient was diagnosed with HIV and started on AZT/3TC/NVP, with a baseline CD4^+^ count of 84 cells/μL
May 2022	The antiretroviral regimen was switched to bictegravir/emtricitabine/tenofovir alafenamide.
June 18, 2022	The patient presented to Tianjin Second People’s Hospital due to mildly enlarged cervical lymph nodes; lymph node biopsy indicated reactive hyperplasia, and PET-CT revealed diffuse lymphadenopathy with increased FDG uptake. While lymphoma was considered in the differential, inflammatory changes were favored.
July 4, 2022	The patient returned with new-onset high fever (39.5 °C), fatigue, anorexia, chest tightness, shortness of breath, and progressively enlarged lymph nodes. Repeat lymph node puncture still indicated reactive proliferation; bone marrow examination showed no abnormal lymphocytes. Metagenomic next-generation sequencing (mNGS) of lymph node tissue detected HHV-8 and EBV sequences. The lymph nodes regressed after anti-infective and glucocorticoid therapy, and the patient was discharged.
August 27, 2022	The patient was transferred to our hospital with recurrent high fever and progressive lymphadenopathy. Laboratory tests showed thrombocytopenia, elevated inflammatory markers, and positive syphilis serology. Imaging revealed splenomegaly and retroperitoneal lymphadenopathy. Excisional biopsy of cervical and inguinal nodes pathologically confirmed HHV-8-positive multicentric Castleman disease (plasma cell type)
September 22, 2022	Treatment with the R-VP16 regimen (rituximab + etoposide) was initiated for MCD, and benzathine penicillin was concurrently administered for latent syphilis.
January 2023	Follow-up PET-CT showed a significant reduction in the size, number, and FDG uptake of lymph nodes throughout the body, indicating a favorable treatment response.Post-treatment RPR titers remained stable at 1:2.
Through August 2025 (36-month follow-up)	The patient has maintained sustained complete remission with no evidence of disease recurrence

## Discussion

The diagnosis of HHV8-positive multicentric Castleman disease (MCD) in people with HIV remains a considerable clinical challenge, often due to its resemblance to other lymphoproliferative disorders or infections—a difficulty clearly illustrated in this case. The patient’s initial presentation with painless lymphadenopathy and reactive lymph node biopsy findings represents a common diagnostic pitfall, as HHV8-MCD can be histologically similar to benign reactive hyperplasia in early disease stages (10). Subsequent emergence of systemic symptoms—including high fever and progressive lymphadenopathy—aligns with the characteristic inflammatory flare of HHV8-MCD, typically driven by HHV8 reactivation and associated cytokine storm (11). The use of metagenomic next-generation sequencing (mNGS) proved pivotal here, enabling detection of HHV8 and EBV sequences and underscoring its growing utility in identifying occult viral infections in cases of atypical lymphadenopathy with non-diagnostic histopathology (12). Although less frequently reported, the co-detection of EBV in HHV8-MCD has been described and may contribute to lymphoproliferative pathogenesis (13).

This case also underscores the diagnostic complexity introduced by co-infections. The concurrent identification of latent syphilis—while not typical of HHV8-MCD—highlights the importance of maintaining a broad differential diagnosis for lymphadenopathy in people with HIV, which should include infectious causes such as syphilis that may present with isolated lymph node enlargement (14). Initial PET-CT findings demonstrated high FDG avidity across multiple nodal stations, raising strong suspicion for lymphoma—a critical and frequent differential in this patient population (15). This imaging phenotype is well-documented in both aggressive lymphomas and HHV8-MCD, underscoring the necessity of histopathologic confirmation (16). The marked treatment response to R-VP16 (rituximab plus etoposide) aligns with established evidence supporting rituximab-based therapy as the cornerstone of HHV8-MCD management, effectively targeting HHV8-infected B-cell reservoirs to achieve remission (17). Nonetheless, the potential long-term risk of progression to HHV8-associated lymphomas—even after remission—remains a concern, as documented in cases of refractory large B-cell lymphoma emerging years after MCD diagnosis (18).

This diagnostic trajectory highlights several key clinical lessons, particularly the histologic mimicry and increasing importance of ancillary testing. The initial biopsy showing reactive hyperplasia is a recognized diagnostic trap, as HHV8-positive MCD may exhibit nonspecific follicular and paracortical hyperplasia that closely mimics benign reactive processes (19). This overlap necessitates a high index of suspicion in people with HIV who have persistent or systemic symptoms, since classic histologic features of MCD—such as expanded mantle zones with “onion-skinning”—may be absent in early biopsies (20). The definitive diagnosis ultimately relied on HHV8 immunohistochemistry, emphasizing that a targeted pathologic evaluation is essential to distinguish this lymphoproliferative disorder from other infectious or autoimmune mimics (21).

From a pathophysiologic standpoint, this case reflects the intricate interplay between viral oncogenesis and host immunity. HHV8-driven overproduction of viral IL-6 and human IL-6 constitutes the central mechanism underlying systemic inflammation and B-cell proliferation in MCD (2). The elevated serum IL-6 level observed in this patient corroborates this pathway. Moreover, the co-detection of EBV via mNGS—though its clinical significance in HHV8-MCD is not fully elucidated—may suggest a cooperative role in lymphomagenesis, as both viruses are associated with B-cell disorders (8). The efficacy of R-VP16 is consistent with a dual strategy: rituximab targets HHV8-infected B cells, while etoposide mitigates cytokine-driven proliferation (22). However, long-term management remains challenging. Some evidence suggests that HIV-negative individuals with HHV8-MCD may experience higher rates of progression after rituximab-induced remission compared to those with HIV, possibly due to differences in immune reconstitution (17). This reinforces the need for vigilant long-term monitoring of HHV8 viral load and inflammatory markers to detect early relapse or progression to lymphoma (23).

In summary, this case highlights the diagnostic and therapeutic complexities of HHV8-positive MCD in people with HIV, particularly when complicated by co-infections such as syphilis. Although the successful response to R-VP16 and penicillin illustrates the effectiveness of targeted treatment, this report also reveals limitations—including its single-patient scope and lack of long-term durability data. The repeated diagnostic challenges, from histologic mimicry to overlapping radiologic features, emphasize the necessity of a multidisciplinary approach incorporating advanced molecular tools such as mNGS and repeated histopathologic review.

Future studies should aim to standardize diagnostic algorithms and optimize treatment protocols for HIV-associated MCD, especially in resource-limited settings with high HHV8 prevalence. Longitudinal research is also needed to clarify the interactions between HHV8, EBV, and other co-infections in the development of lymphoma. This case reinforces the importance of early recognition and personalized management in improving outcomes for this rare yet life-threatening condition.”

## Conclusion

This case illustrates the significant diagnostic and therapeutic challenges of HHV8-positive MCD in people with HIV, where its resemblance to reactive hyperplasia, lymphoma, and other infections necessitates a high index of suspicion and reliance on advanced diagnostics such as mNGS and immunohistochemistry. The concurrent finding of syphilis underscores the need for comprehensive evaluation of co-infections in immunocompromised hosts, while the patient’s response to combined R-VP16 and antibiotic therapy highlights the value of targeted treatment. Nevertheless, the persistent risk of progression to lymphoma emphasizes the importance of long-term monitoring, multidisciplinary collaboration, and further research toward standardized protocols, especially in HHV8-endemic regions.

## Data Availability

The original contributions presented in the study are included in the article/supplementary material. Further inquiries can be directed to the corresponding author.
